# A case-control analysis of common variants in GIP with type 2 diabetes and related biochemical parameters in a South Indian population

**DOI:** 10.1186/1471-2350-11-118

**Published:** 2010-07-30

**Authors:** Divya Sugunan, Anup K Nair, Harish Kumar, Anilkumar Gopalakrishnapillai

**Affiliations:** 1 Amrita School of Biotechnology, Amrita Vishwa Vidyapeetham, Amritapuri PO, Kollam, Kerala, 690 525, India; 2 Amrita Institute of Medical Science, Amrita Vishwa Vidyapeetham, AIMS Ponekkara PO, Kochi, Kerala, 682 041, India

## Abstract

**Background:**

Glucose-dependent insulinotropic polypeptide (GIP) is one of the incretins, which plays a crucial role in the secretion of insulin upon food stimulus and in the regulation of postprandial glucose level. It also exerts an effect on the synthesis and secretion of lipoprotein lipase, from adipocytes, important for lipid metabolism. The aim of our study was to do a case-control association analysis of common variants in *GIP *in association with type 2 diabetes and related biochemical parameters.

**Method:**

A total of 2000 subjects which includes 1000 (584M/416F) cases with type 2 diabetes and 1000 (470M/530F) normoglycemic control subjects belonging to Dravidian ethnicity from South India were recruited to assess the effect of single nucleotide polymorphisms (SNPs) in *GIP *(rs2291725, rs2291726, rs937301) on type 2 diabetes in a case-control manner. The SNPs were genotyped by using tetra primer amplification refractory mutation system-PCR (ARMS PCR). For statistical analysis, our study population was divided into sub-groups based on gender (male and female). Association analysis was carried out using chi-squared test and the comparison of biochemical parameters among the three genotypes were performed using analysis of covariance (ANCOVA).

**Result:**

Initial analysis revealed that, out of the total three SNPs selected for the present study, two SNPs namely rs2291726 and rs937301 were in complete linkage disequilibrium (LD) with each other. Therefore, only two SNPs, rs2291725 and rs2291726, were genotyped for the association studies. No significant difference in the allele frequency and genotype distribution of any of the SNPs in *GIP *were observed between cases and controls (*P *> 0.05). Analysis of biochemical parameters among the three genotypes showed a significant association of total cholesterol (*P *= 0.042) and low density lipoprotein (LDL) with the G allele of the SNP rs2291726 in *GIP *(*P *= 0.004), but this was observed only in the case of female subjects. However this association does not remain significant after correction for multiple testing by Bonferroni's inequality method.

**Conclusion:**

No statistically significant association was observed between any of the SNPs analysed and type 2 diabetes in our population. But the analysis of biochemical parameters indicates that the G allele in rs2291726 may be a putative risk allele for increased LDL cholesterol and further studies in other population needs to be carried out for ascertaining its role in cholesterol metabolism and subsequent cardiovascular risk.

## Background

Type 2 diabetes is a complex metabolic disease, primarily characterised by insulin resistance, relative insulin deficiency and hyperglycemia [1]. According to the Diabetic Atlas 2009 published by the International Diabetic Federation, the prevalence of type 2 diabetes in Indian population is estimated to be around 51 million and India is regarded as the "diabetic capital of the world" [2]. The high prevalence of diabetes in Asian Indians and in particular, South Indian population goes along with higher adiposity, central obesity and high familial aggregation of diabetes [3]. The genetic basis of type 2 diabetes also differs considerably from the western population and efforts are going on to understand the genetic nature of type 2 diabetes in South Indian population [4-6].

It is a well established fact that type 2 diabetes is caused by the interplay of a triad which includes the progressive decline in insulin producing β-cells, an increase in insulin resistance and increased hepatic glucose production [7]. In addition to these, evidences from recent studies also suggest a role for gastrointestinal hormones like incretins which include GIP and glucagon-like peptide -1(GLP-1) towards the manifestation of type 2 diabetes. [8]. GIP is a single 42 amino acid peptide derived from the post-translational processing of a 153 amino acid precursor [9]. It is secreted predominantly by k-cells and released from the upper small intestine (duodenum and proximal jejunum) in response to nutrient ingestion, mainly glucose or fat rich meal [10,11]. This increased level of GIP induces insulin release from the pancreatic β-cells and is responsible for about 70% of postprandial insulin secretion [12]. GIP stimulates glucose-dependent insulin secretion via the activation of their specific GIP receptors (GIPR) expressed on the membrane of pancreatic beta cells. This in turn activates adnenylyl cyclase, phospholipase A and extracellular kinase (ERK and MAP), as a result of which, there is a change in the cellular ion flux ultimately aiding insulin secretion from the pancreatic beta cells [13-18].

In addition to its role in facilitating the release of insulin from pancreatic beta cells, GIP also promotes the proliferation of these cells and prevents their apoptosis hence preventing beta cell dysfunction. [19]. GIP has also been shown to stimulate adipocytes to synthesise and release lipoprotein lipase which hydrolyses lipoprotein associated triglycerides and facilitates its local uptake as free fatty acids into adipocytes [20]. Subsequent studies with GIPR-/- mice revealed that GIP is an obesity promoting factor. [21-23]. This led to the suggestion that GIPR antagonist may be useful in treating type 2 diabetes in European population where it is closely related to obesity and GIPR agonists may give a good indication with diabetes related to impaired insulin release, especially in Asia [24]. Though there is no conclusive evidence in the literature suggesting an altered expression of GIP, it has been shown that, in type 2 diabetes, the overall incretin effect is reduced and this was mainly attributed to altered functioning of GIP [25,26]. The physiological role of GIP in insulin release and fat metabolism combined with the fact that there is a reduction in the incretin effect in type 2 diabetes makes *GIP *and its receptor suitable candidate genes for genetic association studies in type 2 diabetes. Though many studies have been undertaken to analyse the role of common variants in *GIPR *for association with type 2 diabetes, very rarely have these studies looked at the common variants in *GIP*. Inke et al. analysed two SNPs, rs2291725 (G>A, Ser103Gly) and rs2291726 (A>G, a putative splice site SNP) in *GIP*, for association with traits of the metabolic syndrome in a case- control study in European population but failed to observe any significant difference. Further analysis of these two SNPs to analyze the association with type 2 diabetes proved to be negative [27]. However, the sample size used to detect association with type 2 diabetes was small and this study was carried out only in one population. So it is important to use a larger study group and also a different population before ruling out the possible association of GIP with type 2 diabetes.

In this study, we have used a case-control approach for analysing three common variants in *GIP*, rs2291725 (A>G, Ser103Gly), rs2291726 (G>A, intron-exon boundary) and rs937301 (C>T, promoter) using a total of 2000 subjects which includes 1000 (584M/416F) unrelated cases with type 2 diabetes and 1000 (470M/530F) normoglycemic control subjects belonging to South Indian population. We also report association analysis of these SNPs with age of onset of diabetes, Body Mass Index (BMI) and biochemical parameters related to type 2 diabetes.

## Methods

### Patients and controls

Blood samples were collected from a total of 1002 unrelated patients (584 M & 418 F) visiting the outpatient section of the endocrinology department at Amrita Institute of Medical Science (AIMS), Kochi, Kerala during the period 2008-2009. The inclusion criteria of patients for this study were: a) clinically diagnosed as type 2 diabetes, which includes the scrutinizing of medical records for symptoms, use of any medication and measuring the fasting blood glucose levels following the guidelines of American Diabetes Association [28], b) age of onset or diagnosis should be less than 60 years, c) should not have any other metabolic diseases and d) belonging to Dravidian ethnicity. Blood samples of patients from outside Kerala and patients migrating from other parts of the country were not included in the study. Subjects with monogenic forms of diabetes, drug induced diabetes or type 1 diabetes were excluded from the study. All individual and clinical characteristics of the study subjects like age, age at diagnosis, weight, height, body mass index (BMI), smoking and alcohol status, food habits, whether using insulin or not, family history of diabetes, fasting blood sugar, lipid profile and Creatinine values were taken and documented. When available, details of diabetes related complications like diabetic nephropathy, retinopathy and neuropathy were also recorded. A detailed questionnaire was included along with the informed consent form and details of other diseases and medications were taken and documented.

Age, sex and ethnicity matched normoglycemic control subjects were recruited in the study by public advertisement and by offering screening for diabetic risk factors and its awareness, from various parts of Kerala by organizing medical camps. Each of the participants was administered to a health questionnaire which included personal and family history of the subjects, height, weight and blood pressure. Blood pressure was measured after 5 min rest in the sitting position using an automated sphygmomanometer. Fasting blood sugar for all the study participants were checked and documented. A 2 hour plasma blood sugar (PPBS) was also checked for the respective subjects and only those subjects with PPBS < 140 mg/dl and a fasting blood sugar value <110 mg/dl was included in the study. All blood samples were collected under the supervision of an attending physician. From the large number of subjects screened during this study only 1000 (470M/530F) subjects fulfilled the inclusion criteria for normoglycemic controls. All the selected normoglycemic control subjects were thoroughly screened for negative family history of diabetes. More than 500 newly diagnosed diabetic cases were reported during this screening. The inclusion criteria for the healthy controls were: a) should be above 40 years of age, b) should not have any first degree relatives with diabetes, c) should not be taking or have taken any oral hypoglycemic agent or insulin, d) should not have elevated fasting blood sugar level (>110 mg/dl) and a 2 hour post prandial blood sugar level >140 mg/dl. Clinical characterisation of the study subjects is summarized in the Table 1.

**Table 1:** Clinical characterisation of the study population

Characteristics	Patients (T2D)	Controls
n	1000	1000
Sex (Male/Female)	584/416	470/530
Age (years)	55.7 ± 10.2	51.07 ± 10.2
Age of onset of diabetes (years)	45.08 ± 9.04	NA
BMI (Kg/m^2 ^)	25.05 ± 3.4	23.09 ± 3.9
FBS (mmol/l)	8.67 ± 3.4	4.9 ± 0.58
HbA1c (%)	8.3 ± 1.8	-
Triglyceride (mmol/l)	1.63 ± 0.83	-
Total cholesterol (mmol/l)	5.02 ± 1.28	-
HDL (mmol/l)	1.19 ± 0.35	-
LDL (mmol/l)	3.05 ± 1.02	-

n: number of total subjects

Values are expressed as mean ± SD

All the patients and healthy controls were explained the purpose of the study and the complications of investigative procedure. 5 ml peripheral blood samples were collected after they signed the written informed consent. The study was approved by the institutional ethics committee [AIMS, Kochi] following the Indian Council of Medical Research guidelines for handling human samples.

### Genotyping and SNP analysis

Genomic DNA was isolated from patients and healthy volunteers using salting out method [29]. DNA concentration was detected by UV-VIS spectroscopy and diluted to a final concentration of 100 ng/μl. A total of 3 SNPs in *GIP *were selected for the current analysis. rs2291725 and rs2291726 were selected based on a previous study in European population. Additionally one more SNP, rs937301 was selected for the analysis which is a promoter polymorphism with a minor allele frequency >0.1 in the study population. SNPs were genotyped by using tetra primer ARMS PCR [30]. Each PCR reaction was carried out in a total reaction volume of 15 μl. Primer sequences and PCR conditions for the three SNPs have been provided in additional file 1, Table S1. The resultant products obtained after PCR were separated by electrophoresis on 2.5% agarose gel containing ethidium bromide and visualised by gel documentation system (Bio-rad, USA). Allele frequencies for each SNP were calculated by allele counting. Randomly selected 20% of samples were re-genotyped for cross validating initial genotypes. In case of unclear genotyping results, the samples were repeated again in duplicates till clear genotype was available. Unclear genotyping results, even after repetition was excluded from the study. No genotyping error was observed during cross validation. Nucleotide sequence and SNP details were obtained from SNPper http://snpper.chip.org/ and cross validated with the sequence from NCBI http://www.ncbi.nlm.nih.gov/.

### Statistical Analysis

Allele and genotype frequencies were calculated for the whole cohort and analysed for any deviation from Hardy-Weinberg Equilibrium (HWE). HWE analysis was carried out with help of statistical webpage, http://ihg2.helmholtz-muenchen.de/cgi-bin/hw/hwa1.pl. Comparison of allele frequencies and genotype distributions between case and control samples were done by Pearson's chi- square test. Clinical variables such as age of diagnosis, fasting blood sugar, Creatinine and HbA1c were compared using one-way analysis of variance (ANOVA). The study population was divided into two sub-groups based on gender (male/female). The difference in BMI and lipid profile among different genotypic individual were assessed using the analysis of covariance (ANCOVA) to correct for age with respective parameters. P < 0.05 was considered to be statistically significant. All the analysis was performed using the statistical webpage, VassarStat: Statistical Computation http://faculty.vassar.edu/lowry/VassarStats.html. Correction for multiple testing was done by Bonferroni's inequality method wherever applicable.

## Results

### Allele frequency and genotype distribution

Initial genotyping in 300 case samples and an equal number of control samples revealed that the two SNPs, rs2291726 and rs937301 are in complete LD with each other in our study population. So for further analysis only rs2291726 was genotyped. The genotype distributions of the two gene variants in GIP (rs2291725 and rs2291726) did not show significant variation from Hardy-Weinberg proportions. The allele frequency and genotype distribution of both the SNPs in cases and controls have been summarised in Table 2. The allele frequencies for the major and minor allele of rs2291725 were 0.56 and 0.44 respectively in patients as compared to 0.57 and 0.43 respectively in controls (OR = 1.022 [0.902-1.159], *P *= 0.728) whereas, the allele frequencies in the case of rs2291726 for the major and minor allele were 0.53 and 0.47 respectively in patients as compared to 0.53 and 0.47 respectively in controls (OR = 0.998 [0.881-1.130], *P *= 0.974). No significant difference between the genotype groups were observed in both the SNPs (rs2291725 and rs2291726) among cases and controls. We also tested whether there is any significant difference in a dominant or negative model for both the SNPs but failed to observe any statistically significant difference.

**Table 2:** Association analysis of GIP SNPs with Type 2 Diabetes

	rs2291725	rs2291726
Major allele	A	G
Minor allele	G	A
**Controls**		
n	998	1000
Major homozygous	325	297
Hetrozygous	481	472
Minor homozygous	192	231
Major allele frequency	0.57	0.53
Minor allele frequency	0.43	0.47
HWE (P -value)	0.555	0.100
		
**Cases (T2DM)**		
n	989	1000
Major homozygous	326	283
Hetrozygous	458	501
Minor homozygous	205	216
Major allele frequency	0.56	0.53
Minor allele frequency	0.44	0.47
HWE (P -value)	0.060	0.837
**Tests for association (C.I.: 95% confidence interval)**		
Odds ratio	1.022	0.998
Allele frequency	0.728	0.974
Dominant model	0.850	0.490
Recessive model	0.406	0.421

n: total number of subjects

HWE - Hardy-Weinberg equilibrium

### Association analysis with diabetes related biochemical parameters

Association analysis of SNPs in GIP with biochemical parameters was done by using ANCOVA. For association analysis of serum lipid levels only those subjects who were not taking any cholesterol modulating drug based on the health questionnaire during sample collection were included for the analysis. We found a significant association of the SNP rs2291726 with total cholesterol and LDL in female subjects. However this association was observed only in female subjects and did not achieve the desired significance level after correction for multiple testing [*p *= 0.06 (*p *× 15 tests)]. LDL showed a stronger association with the SNP rs2291726 (P = 0.004) than total cholesterol (P = 0.04). Interestingly we did not observe this trend in male subjects and also we did not observe any significant difference when we did a combined analysis (male and female). There was no significant association of the SNP rs2291725 with total cholesterol and LDL in males or females.

We also analysed whether the two SNPs have any role in measures of Fasting blood sugar (FBS) or BMI but failed to observe any significant association. Creatinine values also did not show any significant difference between the three genotypes in both the SNPs. And finally we analysed whether these SNPs play a role in early age of onset of diabetes in this population but did not observe any significant difference between the three genotypes in both the SNPs. The results for association analysis with biochemical parameters has been summarised in Table 3 and Table 4.

**Table 3:** Association analysis of SNPs rs 2291725with biochemical parameters in diabetes

	AA	GA	GG	TOTAL	*P*	P*
**Age of on set of diabetes (years)**	44.64 ± 9.14	45.88 ± 8.77	44.16 ± 9.45	45.13 ± 9.05	0.064	NA
**Fasting Blood Glucose (mmol/l)**	8.77 ± 3.02	8.675 ± 3.76	8.50 ± 3.34	8.67 ± 3.44	0.726	NA
**BMI (Kg/m**^**2**^**)**						
Male	24.26 ± 3.08	24.53 ± 3.37	24.57 ± 2.95	24.46 ± 3.19	0.664	0.671
Female	25.92 ± 3.67	26.08 ±3.41	26.16 ± 3.65	26.04 ± 3.54	0.896	0.905
**Total Cholesterol (mmol/l)**						
Male	4.97 ± 1.22	4.95 ± 1.28	4.98 ± 1.11	4.96 ± 1.23	0.980	1.000
Female	5.28 ± 1.28	5.08 ±1.27	5.02 ± 1.02	5.14 ± 1.22	0.404	0.366
**Triglyceride (mmol/l)**						
Male	1.69 ± 0.91	1.59 ± 0.80	1.64 ± 0.81	1.63 ± 0.89	0.601	0.691
Female	1.56 ± 0.64	1.63 ± 0.78	1.67 ± 0.81	1.62 ± 0.74	0.684	0.779
**HDL (mmol/l)**						
Male	1.19 ± 0.50	1.15 ± 0.34	1.15 ± 0.26	1.16 ± 0.38	0.677	0.684
Female	1.23 ± 0.29	1.23 ± 0.31	1.28 ± 0.25	1.24 ± 0.29	0.607	0.583
**LDL (mmol/l)**						
Male	2.93 ± 0.89	3.09 ± 1.06	3.08 ± 0.94	3.04 ± 0.99	0.344	0.279
Female	3.27 ± 1.03	3.13 ±0.98	3.04 ± 0.81	3.25 ±0.97	0.362	0.318
**HbA1c (%)**	8.44 ± 1.98	8.33 ± 1.78	8.21 ± 1.75	8.34 ± 1.85	0.549	NA
**Creatinine (μmol/l)**						
Male	102.92 ± 45.40	102.12 ± 29.73	109.67 ± 32.24	103.83 ± 35.98	0.238	NA
Female	84.29 ± 24.53	82.06 ± 20.37	84.93 ± 27.29	83.49 ± 23.44	0.684	NA

* *P *value adjusted for age, Number of sample analysed for lipid profile - Male= 411, Female- 250, Creatinine - Male-450, Female- 280. NA: Not Applicable. Datas are expressed as mean ± SD

**Table 4:** Association analysis of SNPs rs 2291726 with biochemical parameters in diabetes

	AA	AG	GG	TOTAL	P	P*
**Age of on set of diabetes (years)**	44.04 ± 8.69	45. 24 ± 8.82	45.02 ± 8.53	44.92 ± 8.72	0.287	NA
**Fasting Blood Glucose (mmol/l)**	8.572 ± 3.23	8.709 ± 3.45	8.61 ± 3.10	8.65 ± 3.31	0.869	NA
**BMI (Kg/m**^**2**^**)**						
Male	24.21 ± 2.98	24.64 ± 3.38	24.29 ± 2.99	24.45 ± 3.19	0.419	0.415
Female	26.31 ± 3.86	26.01 ± 3.46	25.83 ± 3.42	26.02 ± 3.54	0.691	0.719
**Total Cholesterol (mmol/l)**						
Male	5.07 ± 1.15	4.97 ± 1.28	4.86 ± 1.19	4.96 ± 1.23	0.477	0.549
Female	4.89 ± 1.05	5.09 ± 1.29	5.41 ± 1.21	5.13 ± 1.23	0.050	0.042
**Triglyceride (mmol/l)**						
Male	1.68 ± 1.03	1.61 ± 0.81	1.70 ± 0.93	1.65 ± 0.89	0.632	0.533
Female	1.59 ± 0.79	1.67 ± 0.81	1.55 ± 0.55	1.62 ± 0.74	0.538	0.578
**HDL (mmol/l)**						
Male	1.16 ± 0.26	1.16 ± 0.34	1.17 ± 0.51	1.16 ± 0.38	0.990	0.990
Female	1.29 ± 0.25	1.29 ± 0.31	1.24 ± 0.29	1.24 ± 0.29	0.309	0.321
**LDL (mmol/l)**						
Male	3.09 ± 0.99	3.09 ± 1.05	2.84 ± 0.85	3.03 ± 0.99	0.097	0.152
Female	2.89 ± 0.85	3.11 ± 0.98	3.43 ± 0.98	3.16 ± 0.97	0.005	0.004
**HbA1c (%)**	8.288 ± 1.76	8.388 ± 1.83	8.33 ± 1.98	8.35 ± 1.85	0.869	NA
**Creatinine (μmol/l)**						
Male	111.05 ± 37.45	106.75 ± 43.04	99.94 ± 39.25	105.81 ± 40.99	0.119	NA
Female	83.97 ± 27.27	81.58 ± 17.26	85.01 ± 25.48	83.16 ± 22.37	0.507	NA

* P value adjusted for age, Number of sample analysed for lipid profile - Male= 411, Female- 250, Creatinine - Male-450, Female- 280. NA: Not Applicable. Datas are expressed as mean ± SD

## Discussion

In this study, we did a case-control analysis of common variants (rs2291726 and rs2291725) in *GIP *with type 2 diabetes in a South Indian population. After genotyping 1000 type 2 diabetic samples and 1000 control samples, we failed to observe any significant association of these SNPs with type 2 diabetes in our population. An association study in one of the European population also did not reveal any significant association of these SNPs with traits of metabolic syndrome or with type 2 diabetes, though the sample size to detect an association with type 2 diabetes was low [27]. Since the role of GIP in the manifestation of type 2 diabetes seems to be different among Europeans and Asians it is important to replicate association studies of *GIP *in Asian population as well [24].

GIP is known to increase fat uptake by promoting the synthesis and secretion of lipoprotein lipase, which in turn breaks down triglyceride to free fatty acids, readily available for local uptake [20]. Infact it has been seen that mice lacking GIPR did not gain weight and develop adiposity in high fat fed conditions and also utilised fat as the preferred source of energy. This suggests that GIP is an obesity promoting factor [21,22]. At the same time GIP is known to promote the release of insulin upon nutrient ingestion and thus helps in maintaining a proper blood glucose homeostasis [12,31,32]. In Europeans, who are more prone to obesity induced diabetes, blocking of GIP action on adipocytes may be more beneficial whereas it has been suggested that in Asians, GIP agonists may have a beneficial effect due to greater incidences of diabetes with impaired insulin secretion [24]. Though we did not observe any significant association of the studied polymorphisms with type 2 diabetes in our study population, we suggest a detailed genetic analysis of SNPs in *GIP *in other Asian populations as well.

To know more about the genetic role of these variants, we extended our studies to the biochemical parameters associated with type 2 diabetes. Interestingly, our association studies revealed a significant association of the SNP rs2291726 with total cholesterol (*P *= 0.042) and LDL (*P *= 0.004) in females. But this association was not observed in males or during combined analysis (males and females). Sex related association is not a unique phenomenon and there have been studies which report sex dependent association of polymorphism in genes related to cholesterol metabolism suggesting a possible role of sex hormones [33]. Infact it has been shown that estrogen has an important role in regulating serum cholesterol levels and results in a lowering of LDL cholesterol and triglycerides and hence relatively protects younger women from cardiovascular diseases [34]. It is interesting to note that a study by Isken et.al. in mice has shown an interactive role of estrogen and GIP signalling in obesity but the molecular mechanism involved in this interaction still needs elucidation. The study reported that GIPR-/- mice were resistant to ovariectomy induced obesity [35]. It has also been proposed that hormonal therapy in post menopausal women can significantly alter the enteroinsular axis which also involves GIP [35]. GIP has also been shown to play an important role in fat metabolism related diseases like non-alcoholic fatty liver disease (NAFLD) and due to its important role in cholesterol metabolism, an altered expression or function of GIP may play a role in the pathogenesis of cardiovascular diseases as well [36,37]. In our study we observed a significant association (P = 0.004) of LDL cholesterol with the SNP rs2291726 in GIP which is in complete LD with the promoter polymorphism rs937301, but the association was observed only in case of female subjects suggesting a role for sex hormones in this process. Though the association failed to reach the desired significance level after correction for multiple testing (*P *= 0.06), it should be noted that the number of female subjects included in the analysis for LDL levels were comparatively less (n = 250). Hence, we suggest further studies in other population with a higher sample size for the analysis of the role of this variant and LDL cholesterol levels particularly in female subjects. It is also interesting to note that post menopausal women are at a higher risk of cardiovascular diseases. LDL being the bad cholesterol is a major risk factor for cardiovascular disease and our study warrants the need of further association studies in other population and functional studies of GIP in relation to estrogen to better understand its role in cholesterol metabolism and cardiovascular diseases. An earlier study in European population looked at the role of common variants in *GIPR *and *GIP *with cardiovascular diseases. But no significant association was observed between these common variants and cardiovascular diseases. The study did report a positive association of the GIPR SNP with HDL but no results was provided for association analysis of biochemical parameters in diabetes and SNPs in GIP [27].

A recent paper by Juris et al. analysed the expression, metabolism and clearance of GIP in human with renal insufficiency [38]. They observed an increased expression of GIP in cases of renal insufficiency. This led us to analyse the association of these SNPs with serum creatinine levels. We also looked at whether these SNPs are associated with diabetic nephropathy in a study population of 400 subjects comprising of 184 subjects with renal insufficiency and 216 control subjects (data not shown). We did not find any significant association with either serum creatinine or diabetic nephropathy.

## Conclusion

The present study indicates that there is no significant association between the *GIP *SNPs, rs2291725, rs2291726 and rs937301 and type 2 diabetes in a South Indian Dravidian population. The result of association analysis of the biochemical parameters of SNP rs2291726 shows a significant association with total cholesterol and LDL in the female subjects suggesting a role for sex hormones in the process but the association does not remain significant after correction for multiple testing

## Abbreviations

GIP: Glucose-dependent insulinotropic peptide, GLP-1: Glucagon-like peptide-1, HDL: High density lipoprotein, LDL: Low density lipoprotein, VLDL: Very low density lipoprotein, ANOVA: Analysis of variance, ANCOVA: Analysis of covariance, SNP: Single nucleotide polymorphism, PCR: Polymerase chain reaction, HWE: Hardy- Weinberg equilibrium, CI: Confidence interval at 95%, LD: Linkage disequilibrium, SNP: Single nucleotide polymorphism, NAFLD: Non-alcoholic fatty liver disease, ARMS PCR: Amplification refractory mutation system-PCR.

## Competing interests

The authors declare that they have no competing interests.

## Authors' contributions

DS did the sample collection, genotyping, data analysis, statistical analysis, contributed to the study design and prepared the manuscript. AKN did the sample collection, statistical analysis and contributed to the study design. HK provided the case samples and monitored the overall progress of sample collection, GA prepared the study design, critically reviewed the statistical analysis and manuscript and monitored the overall progress of the project. All authors read and approved the final manuscript.

## Pre-publication history

The pre-publication history for this paper can be accessed here:

http://www.biomedcentral.com/1471-2350/11/118/prepub

## Supplementary Material

Additional file 1 **Primer sequence and PCR conditions for amplification of GIP SNPs**. Oligoneucleotide sequence and PCR condition for the allele specific genotyping of three SNPs in GIP. Click here for file
