# Supplementary material for: A case-control analysis of common variants in GIP with type 2 diabetes and related biochemical parameters in a South Indian population
Source: BMC Med Genet. 2010 Jul 30;11:118. doi: 10.1186/1471-2350-11-118 (PMC2920866; doi:10.1186/1471-2350-11-118)
Supplement: Additional file 1 — Primer sequence and PCR conditions for amplification of GIP SNPs. Oligoneucleotide sequence and PCR condition for the allele specific genotyping of three SNPs in GIP. [file 1471-2350-11-118-S1.DOC]

| **Table S1. Primer sequence and PCR condition for amplification of GIP SNPs** | | | | | | |  |  |
| --- | --- | --- | --- | --- | --- | --- | --- | --- |
| SNP | Gene | Method | Primer | Sequence | Location | Annealing | Base pair | Reference |
| rs2291726 | GIP | Tetra-primer ARMS-PCR | Outer forward | CTCACGAGTTCATTCGAGAACAGC | Exon 4 | 68˚C, 30 cycles |  | Ye et al, 2001 |
|  |  |  | Inner reverse | CTCCTCCTTCCTATTAGCTTGCCC |  |  | 546 |  |
|  |  |  | Inner forward | CTCGGGCGCTGGAGCTGGACA |  |  |  |  |
|  |  |  | Outer reverse | CCAGGAGTTTGCTCAATGAAGACC |  |  | 223 |  |
| rs2291726 | GIP | Tetra-primer ARMS-PCR | Outer forward | CTCACGAGTTCATTCGAGAACAGC | Intron 3 | 62.5˚C, 30 cycles |  | Ye et al, 2001 |
|  |  |  | Inner reverse | CTAGGGACACTTGAATCTTTTAAGAC |  |  | 426 |  |
|  |  |  | Inner forward | CCCTCTGCTTTTGGGGTTCCGA |  |  |  |  |
|  |  |  | Outer reverse | CCAGGAGTTTGCTCAATGAAGACC |  |  | 352 |  |
| rs937301 | GIP | Tetra-primer ARMS-PCR | Outer forward | CCACCTGAACTGTGCTAATTGG | 5' UTR | 62˚C, 30 cycle |  | Ye et al, 2001 |
|  |  |  | Inner reverse | CGCGCCTGGCCTACAAGGAT |  |  | 203 |  |
|  |  |  | Inner forward | GTTGCCCTCAGTTAGAAGTGG |  |  |  |  |
|  |  |  | Outer reverse | GTGAACTCGATCTTGGCTCAC |  |  | 284 |  |
